# Transcriptional signatures measured in whole blood correlate with protection against tuberculosis in inbred and outbred mice

**DOI:** 10.1371/journal.pone.0289358

**Published:** 2023-08-03

**Authors:** Sherry L. Kurtz, Patrik Rydén, Karen L. Elkins

**Affiliations:** 1 Center for Biologics Evaluation and Research, Food and Drug Administration, Silver Spring, Maryland, United States of America; 2 Department of Mathematics and Mathematical Statistics, Umeå University, Umeå, Sweden; Centenary Institute, AUSTRALIA

## Abstract

Although BCG has been used for almost 100 years to immunize against *Mycobacterium tuberculosis*, TB remains a global public health threat. Numerous clinical trials are underway studying novel vaccine candidates and strategies to improve or replace BCG, but vaccine development still lacks a well-defined set of immune correlates to predict vaccine-induced protection against tuberculosis. This study aimed to address this gap by examining transcriptional responses to BCG vaccination in C57BL/6 inbred mice, coupled with protection studies using Diversity Outbred mice. We evaluated relative gene expression in blood obtained from vaccinated mice, because blood is easily accessible, and data can be translated to human studies. We first determined that the average peak time after vaccination is 14 days for gene expression of a small subset of immune-related genes in inbred mice. We then performed global transcriptomic analyses using whole blood samples obtained two weeks after mice were vaccinated with BCG. Using comparative bioinformatic analyses and qRT-PCR validation, we developed a working correlate panel of 18 genes that were highly correlated with administration of BCG but not heat-killed BCG. We then tested this gene panel using BCG-vaccinated Diversity Outbred mice and revealed associations between the expression of a subset of genes and disease outcomes after aerosol challenge with *M*. *tuberculosis*. These data therefore demonstrate that blood-based transcriptional immune correlates measured within a few weeks after vaccination can be derived to predict protection against *M*. *tuberculosis*, even in outbred populations.

## Introduction

Despite the worldwide implementation of vaccination with *M*. *bovis* BCG (BCG) against *Mycobacterium tuberculosis* (*M*.*tb*.), *M*.*tb*. is a leading cause of death from any infectious agent. Upwards of 1.5 million people die from tuberculosis annually [1]. While BCG protects children against miliary tuberculosis, protection against pulmonary disease in adults is poor in most populations [2]. Because of this shortcoming, at least fourteen novel vaccine candidates are in development to supplement or replace BCG [3]. However, clinical trials to evaluate vaccines against tuberculosis are lengthy, costly, and complex.

Alternate methods of evaluating vaccine efficacy could streamline pre-clinical evaluation of vaccine candidates as well as clinical trials. Immune correlates, including predictive gene signatures, may be important tools. Previously, we and others demonstrated that an *in vitro* assay using *M*.*tb*.-immune lymphocytes co-cultured with *M*.*tb*.-infected macrophages can serve as a functional correlate of protection [4, 5]. Further, using lymphocytes recovered from this assay, we developed a panel of genes whose relative expression correlated with the degree of protection afforded by a panel of vaccines of different efficacies. Gene expression data, coupled with intramacrophage growth inhibition data, also provided a robust tool for predicting vaccine efficacy [4, 5]. While appealing in its measurement of a relevant function, efforts to simplify the macrophage-lymphocyte co-culture assay concept have met with variable success [6–8].

To complement functional assays, transcriptional signatures derived from primary samples such as blood or purified PBL are an attractive target for development as immune correlates. Human gene expression signatures have been developed to predict progression from people who are latently infected with *M*.*tb*. (LTBI) to active infection (reviewed in [9]. Similar studies performed in various strains of mice and non-human primates support this approach [10, 11].

Such transcriptional signatures can also provide important information about biological responses to infection and point to potential mechanisms of protection. For example, a type I interferon-inducible gene signature developed to discriminate active tuberculosis (TB) infection in people from other diseases opened the door to understanding the important role of type I IFN signaling in host defense against *M*.*tb*. [12–15]. This signature was subsequently recapitulated in transcriptomics studies in mice [10], supporting the relevance of gene expression studies in mice having potential applicability to humans.

The development of genetic signatures to predict vaccine-induced protection is considerably less advanced, especially in people. As recently reviewed, the most advanced current correlates of vaccine-induced protection are based on the magnitude of mycobacteria-specific cellular responses, antibodies, or markers of trained immunity [3]. Studies examining cellular responses in humans and non-human primates (NHPs) have largely relied on the characterization of circulating immune cell subpopulations following vaccination, and gene expression within these specific cell types. Whole blood transcriptomics have received less emphasis [16–18]. One recent study examined gene expression in sequential blood samples taken after immunization with the novel adjuvanted vaccine candidate M72/AS01 [19]. This study generated a gene signature that predicted immune activation, but because no data associated with disease progression were integrated into the analyses, this signature does not yet predict protection. Other studies examined gene transcription in cattle or NHPs after vaccination and related these studies to outcomes after challenge and thus vaccine-induced protection; however, these studies either relied on readouts from a small number of genes or did not generate a signature associated with protection [20, 21]. Collectively, most of these studies relied on *ex vivo* stimulation of purified cells or a small, targeted gene subset and did not use a global, unbiased approach to generate predictive correlates of immunity.

Here, we sought to address this knowledge gap in correlates of protection for tuberculosis vaccines and lack of prognostic tools for vaccine development. The primary goal of the current study was to screen for transcriptional correlates that could be measured shortly after BCG vaccination to predict outcomes after vaccination and challenge. Specifically, we focused on leveraging genetically complex Diversity Outbred (DO) mice to define correlates. DO mice were chosen because they are a heterogenous small animal model whose genetic and phenotypic diversity recapitulates the level of genetic diversity found in humans [22]. The outcomes of BCG vaccination and *M*.*tb*. challenge in DO mice appear to mirror the spectrum of outcomes observed in humans more closely than other small animal models [28].

Due to limitations in repeated longitudinal sampling of individual DO mice and the need for large sample sizes, we first performed a correlate screen in inbred C57BL/6 mice and then translated those findings to outbred DO mice. C57BL/6 have been widely used as an early pre-clinical model for immunity to tuberculosis ([23–26]. Because BCG is the gold standard by which all novel TB vaccines will be compared, this study used BCG as the primary test vaccine for correlates screening. We chose whole blood as a clinically relevant biological sample that would provide a path to translation to humans. We focused on a simple format to directly measure relative gene expression *ex vivo* without further manipulation of samples. We identified novel genes that not only reflect successful vaccination, but also shed light on the components of successful immune responses to *M*.*tb*. The results support further development of transcriptional signatures to aid *M*.*tb*. vaccine development via studies in higher animals and people.

## Materials and methods

### Mice

For these studies, 4-week old male C57BL/6J (C57) mice were purchased from Jackson Laboratories (Bar Harbor, Maine) and entered into experiments when they were 6–10 weeks of age. Female Diversity Outbred (DO) mice were also purchased from Jackson Laboratories, obtained from breeding generations 22–31. All animal studies were performed under protocols approved by the Institutional Animal Care and Use Committee (IACUC) of CBER/FDA and conformed to all relevant U.S. regulatory standards, including the Guide for the Care and Use of Laboratory Animals of the National Institutes of Health. Animal studies were performed under FDA ASP#2011–14. All mice were housed in microisolator cages and were given autoclaved food and water *ad libitum*. Within each experiment, all animals were age matched. During the course of these experiments, any animal observed to be in distress as determined by criteria established in our approved animal protocol was euthanized by CO_2_ inhalation.

### Bacteria and growth conditions

*Mycobacterium bovis* BCG Pasteur (BCG) and *M*. *tuberculosis* Erdman (*M*.*tb*. Erdman) were derived from the mycobacterial culture collection of the Trudeau Institute. To generate laboratory stocks, single vials of *M*.*tb*. Erdman and BCG Pasteur were grown in 7H9 media supplemented with OADC, glycerol, and 0.05% Tween 80 (Difco Laboratories, Detroit Michigan) to mid-logarithmic phase as previously described, then frozen in 0.5 ml aliquots at -70°C until use [27]. A sample from each batch of bacterial stock was subjected to quality control experiments to determine the number of colony forming units (CFU), to confirm typical colony morphologies, and to confirm vaccination efficacy or challenge virulence in mice. To generate heat-killed BCG (HK-BCG), individual vials of BCG were incubated at 100°C for 30 min [4]. Samples from test vials were plated on 7H11 agar to evaluate bacterial killing.

### Vaccinations and bacterial infections

Groups of 5–10 C57 mice were vaccinated subcutaneously with 10^5^ CFU of BCG. Additional groups of C57 mice were vaccinated with HK-BCG. Vaccinated and naïve control mice were challenged aerogenically with *M*. *tb*. Erdman eight weeks after vaccination to assess protection. For each aerosol experiment, a frozen aliquot of *M*.*tb*. was thawed, diluted in PBS to the desired concentration and added to the nebulizer. Mice were challenged over a 30-minute exposure in a Middlebrook chamber (GlasCol, Terre Haute, IN). To determine actual delivered dose, five C57 mice were euthanized 4–5 hours after challenge, and the entire lung for each animal was homogenized and plated to determine uptake CFU. The target dose was approximately 100 CFU delivered to lungs. Replicate experiments of similar design and outcome were performed, to achieve a total of ~115 BCG-vaccinated/*M*.*tb*. challenged DO mice.

### Assessment of bacterial organ burdens and tissue pathology

Bacterial burdens in organs were determined four weeks after infection for C57 mice and 14 weeks after infection for DO mice [28]. Mice were euthanized by CO_2_ inhalation, and organs were removed aseptically and transferred to sterile homogenizer bags containing 5 ml of sterile PBS per organ with 10 μg/ml ampicillin per organ. Organs were disrupted using a Stomacher^®^ (Seward, England). To quantitate CFU, diluted homogenates were plated onto 7H11 plates containing 10% OADC enrichment (Becton Dickinson, Sparks, MD) medium, 10 μg/ml ampicillin, 50 μg/ml cycloheximide, and 2 μg/ml 2-thiophenecarboxylic acid hydrazide (TCH) (Sigma). The addition of TCH to the agar plates was used to selectively inhibit BCG, but not *M*.*tb*., growth [29]. In some experiments, portions of each lung were removed and preserved in 10% formalin. Formalin fixed samples were then sent to American Histolabs, Inc. (Gaithersburg, MD), where the tissues were embedded in paraffin, sectioned at 5 μm, and stained with hematoxylin and eosin (H&E). The Image-Pro Plus software (Media Cybernetics, Rockville, MD) was utilized to assess the level of inflammation present in densitometry scans of each H&E stained image. Lung inflammation was quantitated by assigning areas with dark pink and purple color staining as inflamed [30]. The percentage of dark pink and purple colored areas (compared to light pink and open areas) from a lung section of each mouse was determined by the software and reported as percent lung inflammation per sample.

### Measuring *in vivo* gene expression over time

Groups of 7–10 C57 mice were vaccinated with BCG or treated with PBS as described above, euthanized at selected time points, and blood was collected via cardiac puncture. A 200 μl sample of blood was transferred directly to RNALater. RNA was extracted using from whole blood using the Mouse Ribopure Blood RNA kit (Ambion). The remaining blood was pooled, and PBL were isolated using Lymphocyte Separation Medium according to the manufacturer’s instructions (Cellgro, Manassas, VA). Spleens were removed aseptically and transferred to a petri dish containing PBS with 2% fetal bovine serum (FBS) and pooled for processing. Splenocytes were prepared by pressure disruption, as previously described [4]. After single cells suspensions of PBLs and splenocytes were prepared, remaining red blood cells were lysed using ACK lysis buffer. Aliquots of the cell preparations were stained with trypan blue dye to determine cell viability, and viable cells were enumerated using a hemocytometer. For each tissue, 10^7^ cells were pelleted by centrifugation and resuspended in RNALater, and RNA was purified as previously described using Qiagen RNeasy Kits (Qiagen) [31].

RNA quantification was performed with a Nanodrop spectrophotometer (ThermoFisher Scientific). Equal quantities of RNA per sample were used to generate cDNA via commercially available kits and by following manufacturer’s instructions, as previously described [31]. Semi-quantitative real-time PCR was performed using the ViiA7 sequence detection system (Applied Biosystems) with gene amplification by Taq-Man primer-probe pairs (Applied Biosystems). The average expression levels of the housekeeping genes GusB and GAPDH were calculated in order to normalize the level of mRNA in each sample. For each gene, the change in threshold cycle (ΔC_t_) value was calculated by taking the difference between the C_t_ value of the housekeeping gene average and the C_t_ value of the gene.

### Gene expression profiling by RNASeq

Groups of five C57 mice were vaccinated with BCG or treated with PBS as described above. Cardiac blood was collected at 14 days after vaccination, and RNA was purified. RNA derived from blood was treated with GlobinClear (ThermoFisher Scientific) to deplete globin transcripts (27). All RNA samples were treated with DNase to remove contaminating DNA, and then purified using QiaQuick Spin kits (Qiagen). Purified RNA was quantified using a Nanodrop (ThermoFisher Scientific), and the quality of extracted RNA was verified by Bioanalyzer 2100 (Agilent); all samples had an RNA Integrity Number (RIN) greater than 8.5. RNA prepared from blood from five mice was pooled by group within individual experiments, and then pooled RNA samples from three independent experiments using naïve and BCG-vaccinated animals were subjected to RNASeq analyses. Amplification and labeling of RNA samples were performed by the CBER/FDA Sequencing Core Facility using Illumina TotalPrep RNA Amplification (Applied Biosystems) and using an input of 1 μg of total RNA per sample. Sequencing was performed on a MiSeq PE250 platform (Illumina). The RNASeq data have been deposited in NCBI’s Gene Expression Omnibus and are accessible through GEO Series accession number (GSE212586).

### RNASeq data analyses

We performed RNASeq analyses using the CLC Workbench software package version 20.0.4 (Qiagen). Raw paired-end RNA-seq data were subjected to quality control using “Trim Reads” functions to remove adapter contamination. Reads were aligned to the *Mus musculus* genome Ensembl GRC.m38 (release 86) with default settings. Aligned reads were analyzed to determine differentially expressed genes (DEGs), testing for differential expression due to vaccination using the “Differential Expression for RNASeq 2.4” function by comparing the two groups (naïve and BCG-vaccinated) based on a log_2_ fold change. The analysis package calculated the false discovery rate (FDR), which controls the false positive rate among the DEGs. A volcano plot was generated using the comparison between transcripts in samples from BCG-vaccinated mice to those from naïve mice, with FDR -log_10_ *p*-values. The heat map was created using Euclidian distance measure with complete cluster linkage. Filters were set on the data for a minimum fold change of 1.5 and a *p*-value cutoff of < 0.05.

### Analyses of biological pathways and disease related functions

We performed analyses with Ingenuity Pathway Analysis (IPA, Qiagen) using genes with a minimum of 1.5-fold change and a *p*-value < 0.05, calculated from triplicate independent BCG vs. naïve RNASeq samples. The Core Expression and Bioprofiler analyses were performed with this dataset, which included 814 genes. These comparisons were based on a Z-score algorithm to make predictions about cellular processes, diseases, or cellular functions that are likely to influence gene regulation in the sample dataset. These predictions are based on the white paper, “Downstream Effect Analysis Algorithm White Paper” (http://pages.ingenuity.com/IngenuityUpstreamRegulatorAnalysisWhitepaper.html). IPA considers Z-scores > 2 or < -2 as significantly activated or inhibited, respectively, and overlap *p-*values < 10^−3^ as significantly enriched [32].

### Candidate gene validation

Initial gene candidate validation was performed in C57 mice. The 94 most differentially expressed genes in samples from BCG-vaccinated mice compared to samples from naïve mice, comprising both up and down-regulated transcripts, were selected for targeted analyses using an orthogonal technique, qRT-PCR. Only genes that were differentially regulated in all three replicate sample pairs were included. These genes were used to create a custom ABI TaqMan Array card (Applied Biosytems) that contained gene-specific primer-TaqMan probes, or genes were amplified using the same individual primer-probe sets. Two housekeeping genes, GusB and GAPDH, were included in the custom array card as internal controls. Semi-quantitative real-time PCR was performed using the ViiA7 sequence detection system (Applied Biosystems). qRT-PCR and gene expression normalization were performed as described above. For these validation experiments, groups of five mice were vaccinated with PBS, BCG, or HK-BCG, and were euthanized at 7, 14, 21, 28, and 56 days after vaccination. Validation experiments were repeated two (day 7 and 21) to four (days 14, 28, and 56) times using independent groups of vaccinated mice. Blood and PBL were collected and isolated from individual animals, and RNA was prepared as described above. Additionally, aliquots of blood and PBL from day 56 samples were re-stimulated *ex vivo* with heat-killed BCG [33]. Briefly, 200 μl whole, heparinized blood was mixed with 10 μl cDMEM containing 10^5^ BCG, or 10^6^ PBL in 500 μl cDMEM were mixed with 10^6^ BCG in 500 μl cDMEM; all were transferred to individual wells in a 24-well tissue culture plate and incubated overnight at 37C/5% CO_2_. These samples, designated day 56RE, were then processed for RNA purification as described above. For subsequent gene candidate testing using DO mice, blood samples were collected by tail vein nick 14 days before and 14 days after subcutaneous vaccination with 10^5^ BCG Pasteur. Blood was collected directly into RNALater, and blood RNA processing and qRT-PCR were performed as described above.

### Statistical analyses for C57 mice

Data from four time points from C57 mice were considered in the modelling: day 14, 28, and 56 after vaccination, and re-stimulated samples from day 56 (d56 RE) after vaccination. For each time point, 8 mouse groups were treated with BGC (positive samples) and 16 groups were treated with either HK-BCG or PBS (negative samples). The binary response (positive or negative sample) was modelled using linear discriminate analysis (LDA), with a classical setting assuming homoscedasticity and no prior, and with a set of the log_2_-transformed qRT-PCR expression data from 18 select genes as explanatory variables. Cross validation was used to predict the sample-treatment as either positive or negative. The analyses were made using the functions *discrim* and *crossvalidate*.*discrim* in S-plus 8.2 (TIBCO Spotfire). For each LDA analysis, the predicted treatment was compared to the true treatment. Sensitivity, specificity, and accuracy were estimated as the fraction of correctly classified BGC-treated samples, the fraction of correctly classified HK-BCG and the fraction of correctly classified samples respectively.

For each time point, 987 LDA analyses were conducted; 18 models had a single gene predictor, 152 models had two predictors, and 817 models had three genes as explanatory variables. LDA-models with a sensitivity and a specificity above 0.7 were considered good. In order to quantify the genes’ overall ability to predict the treatment the number good models, for which the gene was a predictor, were counted. For a given time point, each gene was included in 138 models.

### Disease evaluation, survival, and statistical analyses for DO mice

Control and BCG-vaccinated DO mice were aerogenically challenged as described above eight weeks after BCG vaccination with ~100 CFU. DO mice were followed for survival for 14 weeks after challenge. Body weight was tracked weekly for each animal beginning at the day of *M*.*tb*. challenge and throughout the course of the experiment, and the percent of weight loss from peak body weight was determined for each animal. Surviving animals were euthanized at 14 weeks after challenge, and lung and spleen *M*.*tb*. burdens and lung inflammation were determined as described above. DO mice chosen for blood transcriptional analyses were a subset of a larger group of ~ 1000 animals from a separate study (Kurtz *et al*. [unpublished]). Animals were ranked based on lung and spleen CFU and survival, and animals with the highest and lowest CFU burdens, as well as those that succumbed to infection before 14 weeks were chosen. Blood RNA was prepared, and qRT-PCR was performed as described above. For the present studies, we obtained gene expression data from 107 DO mice. Lung and spleen *M*.*tb*. burdens, weight loss, and lung inflammation data were included in statistical analyses. The difference in gene expression between measurements observed before (B) and after (A) vaccination were tested using the Wilcoxon rank sum test. Measurements below the considered detection level 0.0005 were set to 0.0005. We expected survival to be associated with a vaccine-induced increase in gene expression and defined the predictors as

$$D=max\left({log}_{2}\left(A/B\right),0\right)$$

The binary response (survived or did not survive) was modelled and evaluated using LDA and cross-validation, as described above. Models with single genes as predictors and models with two or three genes were considered. The models’ sensitivity and specificity were estimated as the fraction of correctly classified survivors and the fraction of correctly classified non-survivors, respectively. In addition, the difference in gene expression between survivors and non-survivors were observed for the log-difference D and the gene expression observed after vaccination (*i*.*e*., A). Wilcoxon rank sum test was used to calculate the corresponding *p*-values.

The pairwise correlation between each of the measurements of lung CFU, spleen CFU and lung inflammation and the genes observed for A- and D-values were obtained using Spearman’s rank correlation.

## Results

### Time course of gene expression and genome-wide screening in whole blood of BCG-vaccinated mice

**Fig 1:**
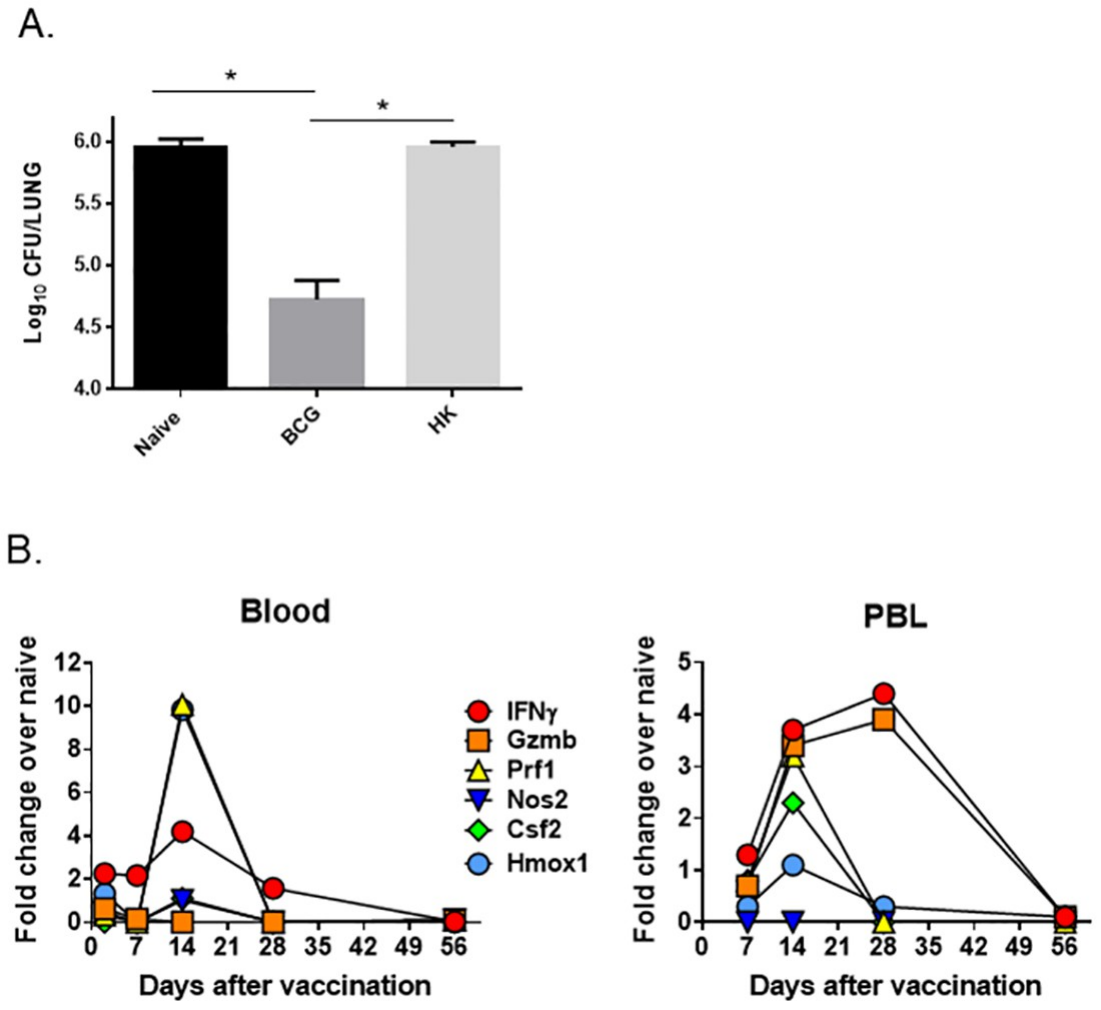
Live BCG protects C57BL/6 mice against *M*. *tuberculosis* aerosol challenge and induces expression of a subset of immune-related genes in blood over time. A) Groups of five C57 mice were intradermally vaccinated with 10^5^ CFU of BCG (BCG), heat-killed BCG (HK-BCG), or PBS (naïve) as a naïve control group. Mice were aerogenically challenged with ~ 100 CFU of *M*.*tb*. eight weeks after vaccination and euthanized 4 weeks after challenge to enumerate *M*.*tb*. CFU. Data presented represent the mean log_10_ *M*.*tb*. burden ± SD in lungs. Results shown are from one representative experiment of three of similar design and outcome. B) Groups of three C57 mice were intradermally vaccinated with 10^5^ CFU of BCG or PBS as a naïve control group. Mice were euthanized at the indicated times after vaccination, and cardiac blood and PBL were collected. RNA from blood or PBL for each group was pooled and assessed by qRT-PCR for the expression of *ifng*, *gzmb*, *prf*, *nos2*, *csf2*, and *hmox1*. The relative expression of each gene in BCG samples compared to naïve (PBS) samples was calculated. The average fold change for single representative of three independent experiments is represented. * p < 0.05 by Student’s *t* test.

While the goal of this study was to explore predictive correlates using DO mice, the heterogenous nature of these animals means that performing a primary screen requires a prohibitively large sample size. The small size of the animals also precludes multiple longitudinal samplings on individuals, limiting time course studies. Therefore, we chose C57BL/6 (C57) mice to perform initial screening and time course studies. We used BCG vaccination of C57 mice to search for genes whose relative expression in blood shortly after vaccination was related to *in vivo* protection. As previously demonstrated [4, 5], C57 mice vaccinated with 10^5^ BCG intradermally, challenged with *M*.*tb*. Erdman 8 weeks after vaccination and evaluated four weeks after challenge contained ~ 1 log_10_ fewer *M*.*tb*. in lungs compared to naïve mice (Fig 1A). In inbred C57 mice, this level of protection is moderate, relatively uniform across animals, and highly reproducible. Mice vaccinated with heat-killed BCG (HK-BCG) and challenged with *M*.*tb*. had significantly larger numbers of *M*.*tb*. in lungs than mice vaccinated with BCG and that were similar to those naïve mice (Fig 1A). HK-BCG was therefore used to represent a “vaccine” that provides little or no protection but will elicits some inflammatory responses, thus aiding to differentiate non-protective inflammation from protective vaccination. The hierarchy of protection against *M*.*tb*. challenge was therefore BCG > HK-BCG ≈ PBS treatment. We used this hierarchy to then evaluate relative gene expression in blood.

To evaluate the time after vaccination when gene expression changes in blood were most robust and thereby derive a single prognostic time point for sampling in the DO mice, we vaccinated C57 mice with BCG or PBS and collected blood 3, 7, 14, 28, and 56 days later. We prepared mRNA and performed qRT-PCR to measure the expression of a subset of immune-related genes. We chose *ifng*, *prf*, *nos2*, *hmox1*, and *csf2* for initial studies based on previous *in vitro* co-culture studies that studied BCG-immune splenocytes and PBLs re-stimulated by co-culture with *M*.*tb*.-infected macrophages [4]. We previously demonstrated that the expression of these genes as measured in lymphocytes recovered from the co-culture was linked to the degree of protection afforded by a panel of vaccines with different efficacies. Here, we calculated the fold change for each gene by comparing expression in samples from BCG-vaccinated mice to samples from PBS-treated mice. In RNA prepared from whole blood, the most robust expression across all genes tested was found at 14 days after vaccination (Fig 1B). Under these conditions, *Hmox1* and *prf1* were the most highly induced genes in whole blood, followed by *ifng*, while *nos2*, *csf2*, and *gzmb* were not upregulated by vaccination. Additionally, we purified PBL from whole blood at 7, 14, 28, and 56 days after vaccination, prepared RNA, and compared gene expression in these purified cells. In PBL, *ifng* and *gzmb* were most induced by BCG vaccination, with expression elevated at day 14 and day 28. Expression of *prf1*, *csf2*, *and nos2* peaked at 14 days and waned by day 28, and *nos2* expression was not increased by vaccination (Fig 1C). Thus, gene expression in whole blood and purified PBL peaked at 14 days after vaccination in most (although not all) genes in this panel. Because this initial set of genes was chosen based on studies that included memory effector T cells in *in vitro* co-culture assays [4], it is not surprising that only some of these genes would be upregulated in blood at these early time points after primary vaccination.

**Fig 2:**
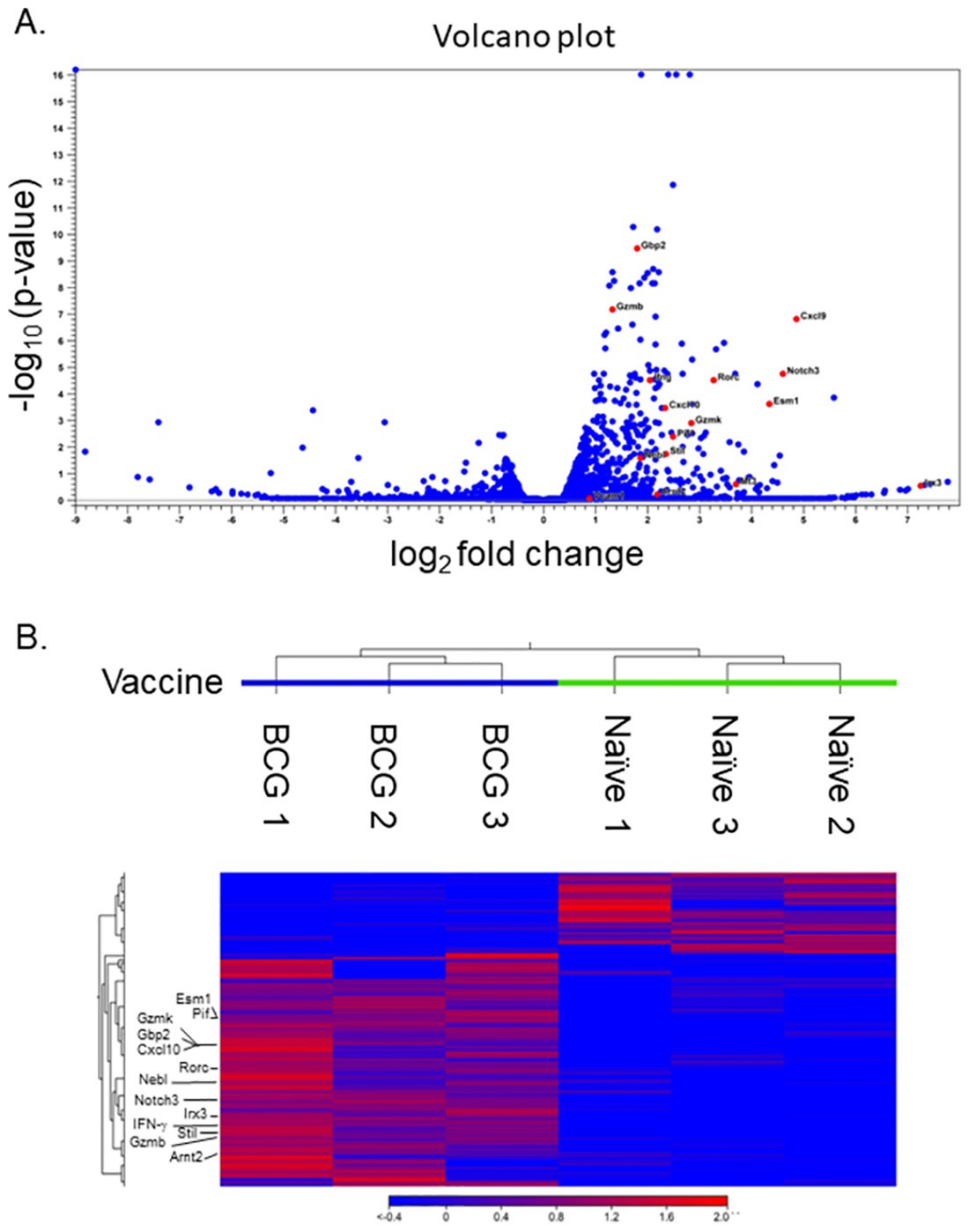
RNASeq analyses identify genes in mouse blood whose expression is induced by BCG vaccination. Groups of three C57 mice were intradermally vaccinated with 10^5^ CFU of BCG or PBS as a naïve control group. Mice were euthanized 14 days after vaccination, cardiac blood was collected for RNA isolation, and RNA was pooled from mice within each group. This experiment was repeated three times, and three sets of pooled BCG-vaccinated and naïve RNA samples were submitted for RNASeq. Gene expression was evaluated by comparing samples from BCG-vaccinated and naïve mice. (A) Differentially expressed genes are visualized by volcano plot, where the magnitude of expression difference is presented (log_2_ ratio) on the X-axis compared to the significance (-log_10_ FDR *p*-value) on the Y-axis for each gene, reflecting expression changes between BCG-vaccinated and naïve mice. Representative genes that were chosen for downstream analyses are shown in red dots and labeled with their gene names. (B) The heat map illustrates the log_2_ fold change up (red) or down (blue) for the top DEG, as selected by genes from RNASeq analyses with a minimum fold change of 1.5 and with *p* < 0.05. Cluster relationships based on Euclidian distance among samples from three independent experiments are annotated. Representative genes that were chosen for downstream analyses are labeled with their gene names.

Based on this time course, as well as the interest in deriving biomarkers that could be used quickly after vaccination, we chose 14 days after vaccination to perform a transcriptome-wide screen for genes induced after vaccination. We focused on blood because of its accessibility in clinical studies and again performed this screen in C57s because of the large numbers of outbred DO mice that would be needed otherwise. We collected whole blood samples from groups of C57 mice at 14 days after vaccination with BCG or treatment with PBS (control), isolated RNA, and pooled RNA from mice within groups in the same experiment. These experiments were repeated three times, and pooled samples from each experiment were submitted for RNASeq analyses. Analyses identified a total of 1198 genes were differentially expressed between BCG-vaccinated and naïve samples with *p*-values < 0.05, and 235 genes with an FDR cutoff of 0.05. We also noted that most differentially expressed genes (DEGs) were upregulated in samples from BCG-vaccinated mice compared to those from naïve mice; few were downregulated. DEGs were visualized using a Volcano plot, where genes were plotted based on log_2_ fold change in expression between BCG-vaccinated and naïve samples in relationship to the -log_10_ FDR *p*-value (Fig 2A). Genes that became the focus of subsequent analyses are labeled to highlight these in the volcano plot. Expression data from DEGs were also plotted as a heat map based on Euclidian distance, using a cutoff for genes of at least 1.5-fold change and *p*-values of 0.05 (Fig 2B). The heat map illustrates clustering of responses in samples within the naïve and BCG-vaccinated groups as well as the differential gene expression between these samples, with genes of downstream interest denoted.

**Fig 3:**
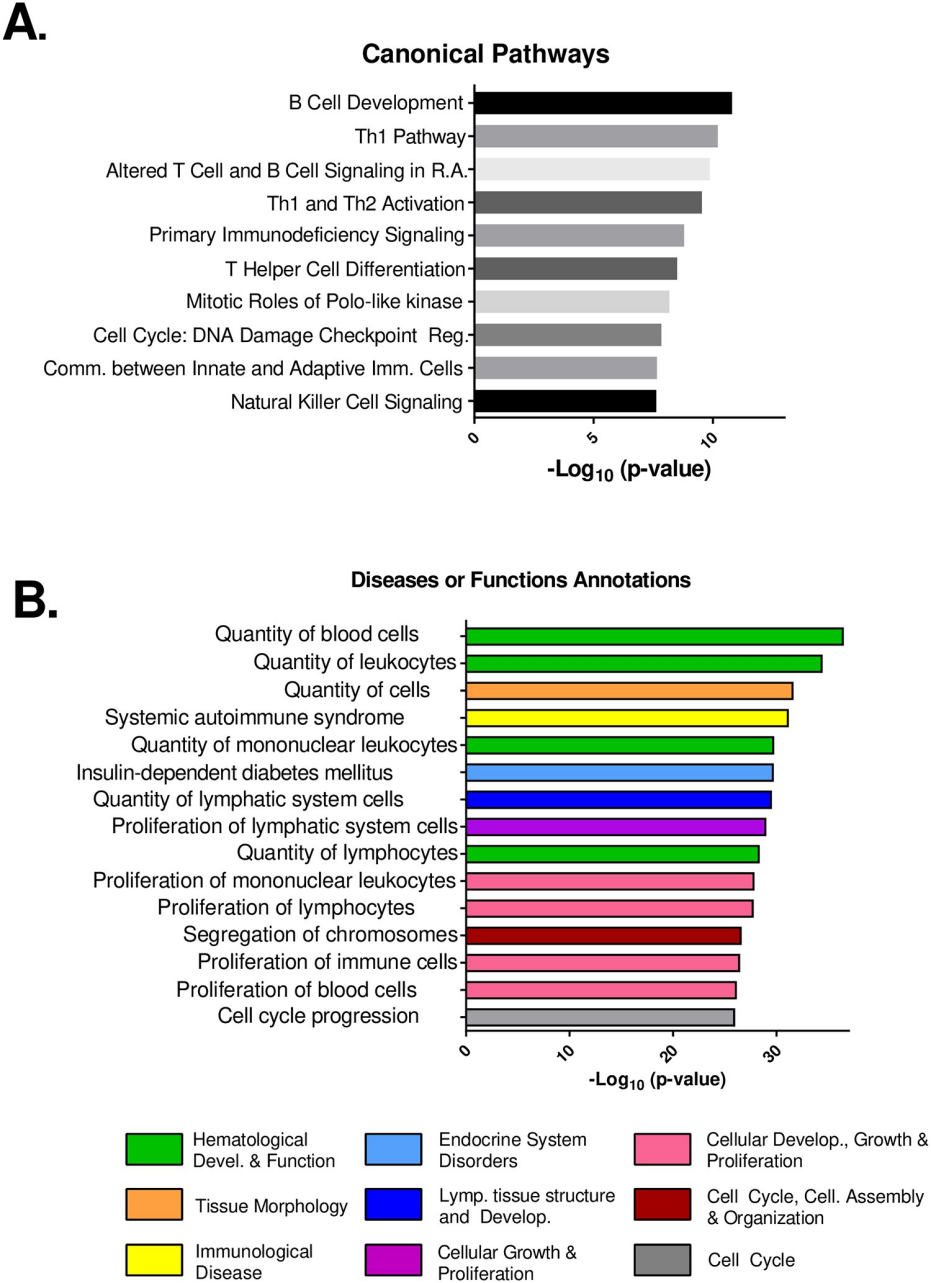
RNASeq analyses of transcriptional changes induced by BCG vaccination reveal canonical biological pathways associated with protection. Using transcriptional expression data derived from RNASeq, we performed analyses with Ingenuity Pathway Analysis software (IPA, Qiagen). Analyses focused on genes with a minimum of 1.5-fold change by RNASeq and a *p*-value < 0.05, calculated from triplicate independent samples from BCG vs. naïve (PBS) mice. This dataset included 814 genes. A) Core Expression analyses revealed canonical biological pathways predicted to be influenced by changes in gene expression. Bars depict the -log_10_ *p*-value for each canonical pathway, based on the number of representative genes in that pathway and the strength of the transcriptional changes. B) Bioprofiler analyses revealed disease associations or functional biological pathways predicted to be influenced by changes in gene expression. Bars depict the -log_10_ *p*-value for each disease or functional subset based on the number of representative genes in that pathway and the strength of the transcriptional change. Colors indicate the biological process superfamily that incorporates the noted individual pathways.

Based on this set of DEGs, we next performed additional analyses to interrogate related biological functions. We used Ingenuity Pathway Analysis (IPA) software to analyze the subset of the DEGs with a cutoff of a ± 1.5-fold change and *p* < 0.05. We first examined the canonical pathways linked to this subset of genes. Genes involved in B cell development and various Th1 signaling pathways were the top sets altered due to BCG vaccination (Fig 3A). Additional pathways upregulated in response to BCG vaccination included cellular proliferation, cell death, innate-adaptive interactions, and NK cell activation (Fig 3A). We performed further analyses to associate gene expression changes with related disease or functional annotation clusters. Several broad categories of disease were associated with vaccination, including “Hematological Development and Function” (Fig 3B). Within this category, we found subsets of functional pathways related to the quantity of blood cells, leukocytes, and lymphocytes that were altered by vaccination with BCG. The category represented by “Cellular Development, Growth, and Proliferation” was also altered by vaccination, including the proliferation of immune cells, lymphocytes, and mononuclear leukocytes. These analyses therefore detected a wide array of biological functions, and the validity of the approach was supported by detection of relevant genes and pathways that are plausibly linked to vaccination.

### Orthogonal validation of gene expression in blood of protected BCG-vaccinated mice compared to non-protected HK-BCG-vaccinated mice

From this initial RNASeq screen, we chose 94 of the most highly differentially regulated genes to generate a list of promising candidates for secondary validation. We focused on genes that were differentially regulated in samples from vaccinated animals in each of the three RNASeq experiments and that represented the different biological pathways described above. In our studies, the most consistently differentially genes were those upregulated by vaccination, and not down-regulated or repressed. We then performed new experiments in which groups of animals were vaccinated with BCG, PBS, or with HK-BCG. Given that BCG is likely to induce inflammatory responses that may not be directly linked to protection, we included HK-BCG as a vaccine that would still contain some of inflammatory properties, such as those stimulated by mycobacterial lipids, but does not provide protection (Fig 1A) and thus serves as an additional negative comparator for protection. In these studies, we collected whole blood and purified PBL at 7, 14, 21, 28, and 56 days after vaccination to assess the time course of expression of the candidate genes (without further sample manipulation). In addition, we re-stimulated day 56 whole blood and PBL *ex vivo* with HK-BCG to activate memory recall responses in these samples.

**Fig 4:**
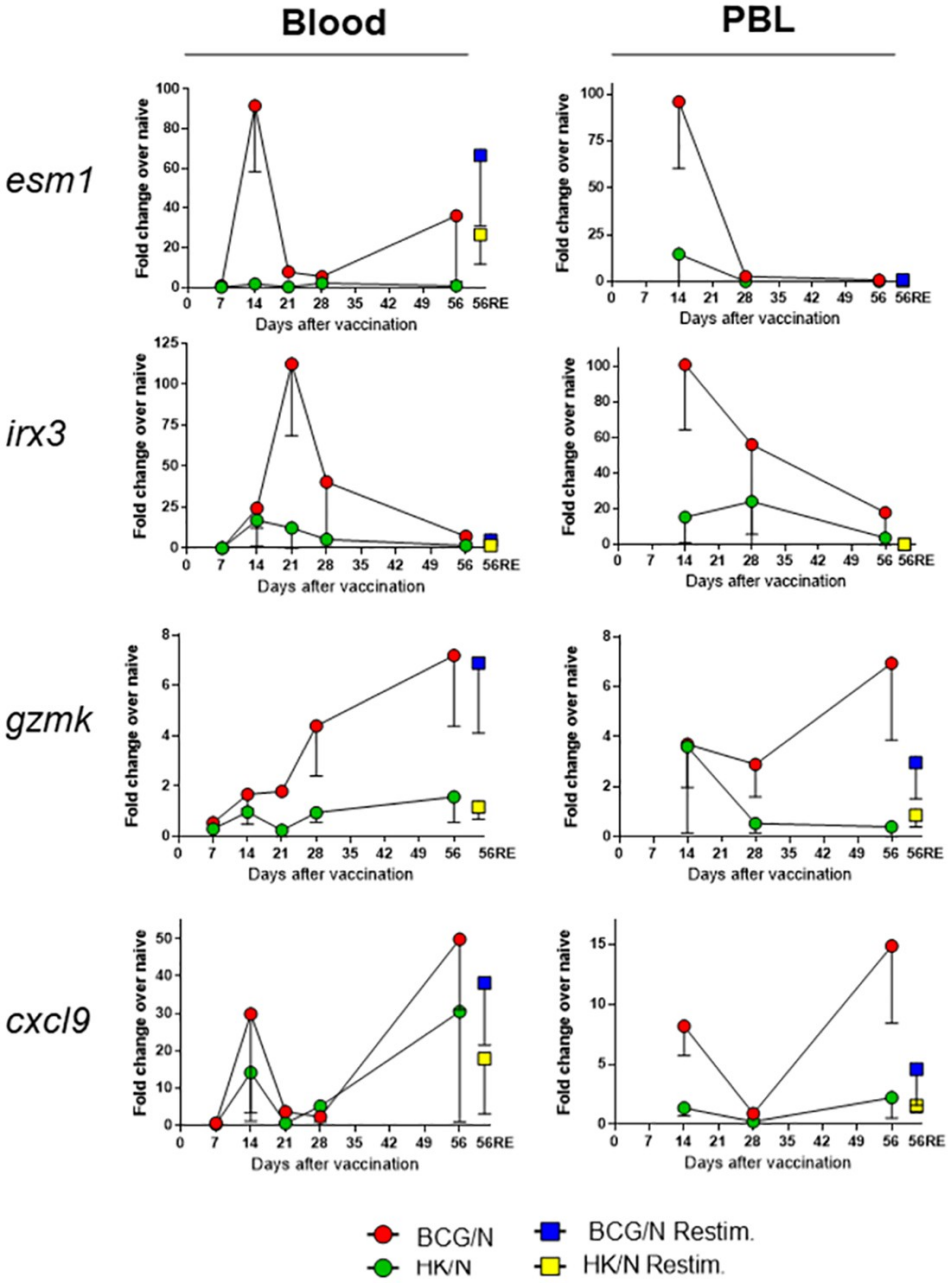
Independent validation of a subset of genes by qRT-PCR demonstrates differential expression in blood and PBL between protective versus non-protective vaccination conditions. Groups of five mice were vaccinated with PBS, BCG, or HK-BCG, and were euthanized at 7, 14, 21, 28, and 56 days after vaccination. Blood and PBL were collected and isolated from individual animals, and RNA was prepared as described. Additionally, aliquots of blood and PBL from day 56 samples were re-stimulated *ex vivo* with heat-killed BCG (56RE). From the RNASeq results, 94 of the strongest gene candidates were chosen for re-screening by qRT-PCR across the time course. Of these 94 genes, expression results for the top four candidates in blood and PBL are depicted: *esm1*, *irx3*, *gzmk*, and *cxcl9*. These experiments were repeated two (day 7 and 21) to four (days 14, 28, 56, and 56RE) times using independent batches of vaccinated mice. Data represent the average and standard deviation (half error bars) for experimental replicates at each time point.

Samples from 5 animals per group from 2–4 independent experiments were used to quantitate relative expression of these candidates by qRT-PCR in whole blood and in PBLs (Fig 4). We calculated the fold change of gene expression in samples from BCG-vaccinated animals compared to HK-BCG-vaccinated or sham PBS-vaccinated animals. We then focused on identifying genes whose expression was robust and consistent in BCG-vaccinated samples compared to samples from HK-BCG-vaccinated or PBS-treated mice; where the time course of expression reinforced robust expression; and where the hierarchy of expression in which BCG > HK-BCG ≈ naïve was reproducible over multiple independent experiments. These criteria narrowed the candidates to a group of 18 genes (Table 1). *Esm1*, *irx3*, *gzmk*, and *cxcl9* were among the most highly DEGs in both blood and PBL. Each, however, had a unique expression pattern. In blood, induction of *esm1* and *cxcl9* expression was highest at 14 days after vaccination and waned by 28 days (Fig 4, left panels). Interestingly, expression of these genes increased again at 56 days, even without re-stimulation. *Irx3* expression was robust in whole blood from BCG-vaccinated animals compared to HK-BCG- or PBS-treated animals, peaking at 28 days and waning thereafter, while *gzmk* expression increased over time and peaked at day 56. *Ex vivo* re-stimulation of whole blood at day 56 induced further increased expression of *esm1* compared to resting day 56 levels, although increases were not evident for *irx3*, *gzmk*, or *cxcl9*.

**Table 1:** Top gene candidates subjected to statistical analyses have high accuracy as single-gene classifiers.

Gene	Day 14	Day 28	Day 56	Day 56RE
***esm1***	***0*.*783*** ^***a***^	***0*.*783***	0.478	***0*.*750***
***cxcl9***	0.609	0.125	0.609	***0*.*708***
***arnt2***	0.696	0.458	0.565	0.583
***cxcl10***	0.261	0.542	0.130	***0*.*708***
***gbp2***	0.681	0.542	0.652	***0*.*75***
***gzmb***	0	0.160	0.087	0.542
***gzmk***	0.609	***0*.*750***	0.434	***0*.*875***
***ifng***	0.521	0.625	0.609	0.625
***irx3***	***0*.*826***	0.583	0.521	0
***mt3***	0.565	0.458	0.652	0.500
***nebl***	0.260	0.417	0.609	0.583
***notch3***	0.565	0.584	0.609	0.666
***pbk***	0.565	0.417	0.652	0.583
***pif1***	0.521	0.666	0.609	0.609
***rorc***	0.608	0.291	0.652	0.583
***stil***	0.636	0.521	0.565	0.417
***syngr4***	0.363	0.541	0.565	0.304
***vcam1***	0.363	0.521	0.087	0.083

Gene expression data for 18 individual genes measured using whole blood were subjected to statistical analyses to determine the accuracy of each individual gene to correctly classify a vaccine group by protective efficacy, *i*.*e*., to classify as belonging to the group vaccinated with BCG versus groups treated with HK-BCG or PBS (naïve).

^***a***^ The accuracy of each gene is listed for day 14, 28, 56, or day 56 re-stimulation (RE) samples. Classifiers that reached a significant 0.7 cutoff for any given time point are highlighted in **bold/italics** text.

The differential expression of these genes was also robust in purified PBL and exhibited similar time courses (Fig 4, right panels). *Irx3* and *esm1* were the most highly differentially expressed genes in PBL; expression peaked at 14 days after vaccination and then waned over time. However, *gzmk* and *cxcl9* expression peaked at 56 days. *Gzmk* was induced at 14 days after vaccination by both BCG and HK-BCG, but expression remained elevated in samples from BCG-vaccinated mice over 56 days and waned in HK-BCG-primed PBL. *Ex vivo* re-stimulation of PBL did not augment differential expression compared to the day 56 levels, but expression of for *gzmk* and *cxcl9* was still elevated in samples from BCG-vaccinated animals versus those from HK-BCG-vaccinated animals. Importantly, patterns of expression were generally similar in PBLs compared to those in whole blood. These observations support the role of leukocytes in driving detection of differences between the vaccine treatments, as well as support pursuing the simpler approach of using whole blood. Altogether, orthogonal validation testing supports the conclusion that extensive screening identified a subset of genes that exhibits robust upregulation in blood and PBLs after successful vaccination with BCG, but not following HK-BCG vaccination or PBS treatment. This hierarchy further provides a first step in relating gene expression induction not just to exposure to live BCG but to successful protection.

### Construction of multivariate models that predict successful vaccination with high sensitivity and specificity

**Fig 5:**
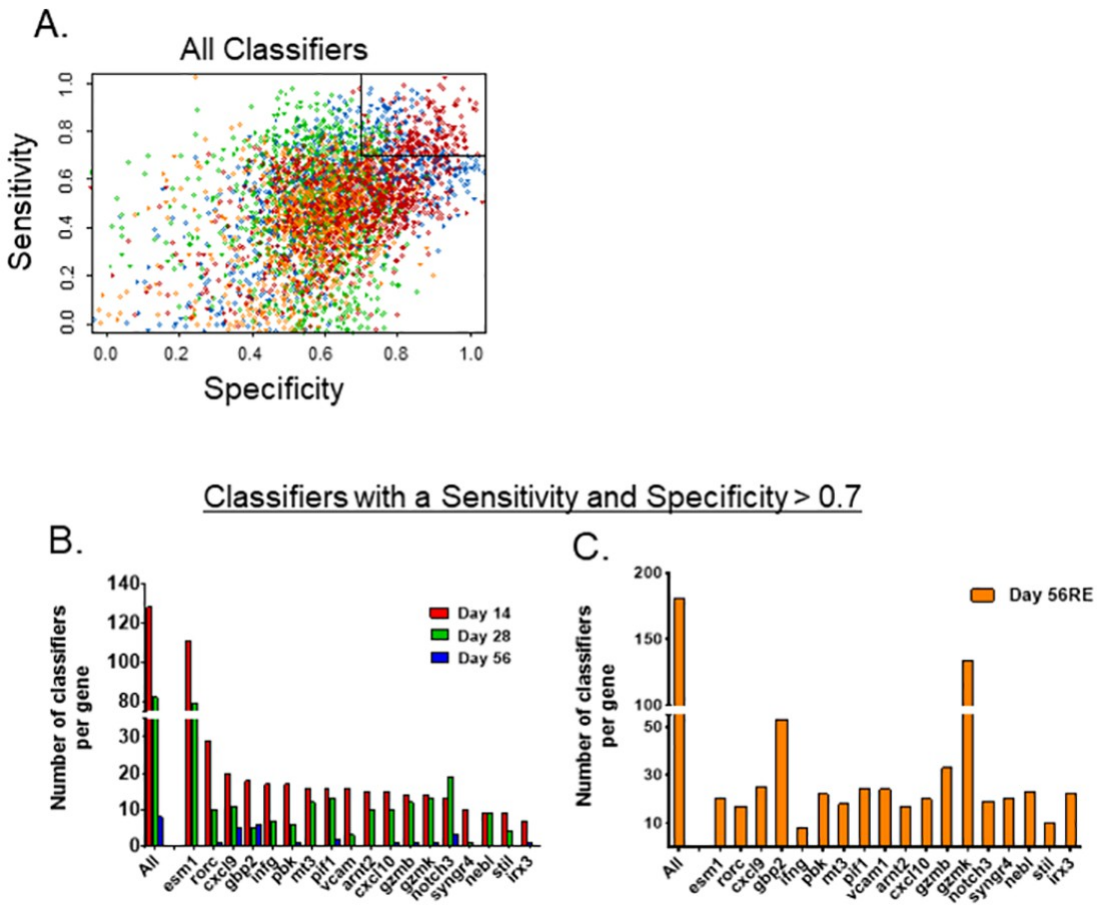
Selected gene classifiers exhibit strong sensitivity and specificity. A) The specificity and sensitivity for all classifiers from blood transcriptional analyses considered are shown for day 14 (blue), day 28 (green), day 56 (orange), and 56 re-stimulated (red). For each time point, 987 classifiers were constructed, of which 18 included one variable (*i*.*e*., one gene), 153 included two variables, and 816 included three variables. 401 classifiers with both sensitivity and specificity above 0.7 were considered to be good (upper right boxed region). B) The bar graphs depict the number of good classifiers (*i*.*e*., classifiers with both sensitivity and specificity above 0.7), in which each gene was an explanatory variable in the classifier for the indicated time points (B) day 14 (red), day 28 (green), day 56 (blue), and (C) day 56 re-stimulated (56RE, orange).

The qRT-PCR data from whole blood were then evaluated for the ability of individual gene expression values, or a combination of values from two or three genes, to correctly discriminate BCG-vaccinated from HK-BCG-vaccinated or sham PBS-treated animals. The goal of these analyses was to use gene expression information to quantitatively predict protective outcomes. We evaluated these genes or combinations of genes, termed ‘classifiers’ here, for sensitivity, specificity, and accuracy of discrimination of vaccine groups. Altogether, we constructed 987 classifiers for each of the four time points (day 14, 28, 56, and day 56 re-stimulated [day 56RE]), and we then focused on classifiers with sensitivity and specificity thresholds above 0.7, designated as “good,” which included 401 classifiers (Fig 5A, inset box). The accuracy of these classifiers was calculated as the fraction of samples correctly classified. Very few genes reached the 0.7 cutoff as single gene classifiers (Table 1). *Esm1* expression levels differentiated BCG from HK-BCG and PBS in samples obtained on days 14 and 28, and in the day 56RE samples, thus separating potentially protective responses induced by BCG from non-specific, non-protective inflammatory responses from HK-BCG. *Irx3* predicted protection at day 14 with high accuracy and *gzmk* at day 28 (Table 1), but neither were successful predictors at other time points. Five single genes, *esm1*, *cxcl9*, *cxcl10*, *gbp2*, and *gzmk*, had predictive accuracies above 0.7 for day 56RE.

Overall, the number of classifiers containing one, two, or three genes with high sensitivity and specificity decreased over time, with 128 classifiers observed at day 14, 82 at day 28 and just 8 at day 56 (Fig 5B). The re-stimulated day 56RE samples comprised 183 good classifiers, a substantial increase over unstimulated day 56 samples (Fig 5C). When evaluated as a component of any significant classifier, *esm1* was present in the majority of classifiers at day 14 and day 28 but was only a minor contributor at day 56RE; *esm1* was not present in any significant classifiers at day 56. *Gbp2* was a major component of good classifiers at several time points, including 75% at day 56 and 29% at day 56RE. Other major contributors to good classifiers were *cxcl9*, *notch3*, *pbk*, and *pif1* at day 56, and *gzmk*, which was present in 73% of day 56RE classifiers.

For any given time point, multi-gene classifiers generally had a higher sensitivity and specificity for predicting protection than any individual gene. The most predictive classifiers at day 14 were 3-gene classifiers that contained combinations of *esm1*, *irx3*, *rorc*, *pbk*, or *cxcl9* (S1 Table). At day 28, the highest performing classifiers included 2 and 3 gene classifiers comprised of combinations of *esm1*, *gbp2*, *notch3*, *cxcl9*, and *cxcl10*. Only 3-gene, not single or 2-gene, classifiers had sensitivity and specificity > 0.7 at day 56, and all of the top five classifiers included *cxcl9*; most included *gbp2*. The top performing classifiers at day 56RE were also 2- or 3-gene classifiers, with *gzmk* and/or *gzmb* present in all of the top 30 combinations. Across all timepoints, each of the 18 genes measured had significant predictive capacity, either as a single gene or as a component of multi-gene classifiers.

### Validation of transcriptional correlates in outbred DO mice

**Fig 6:**
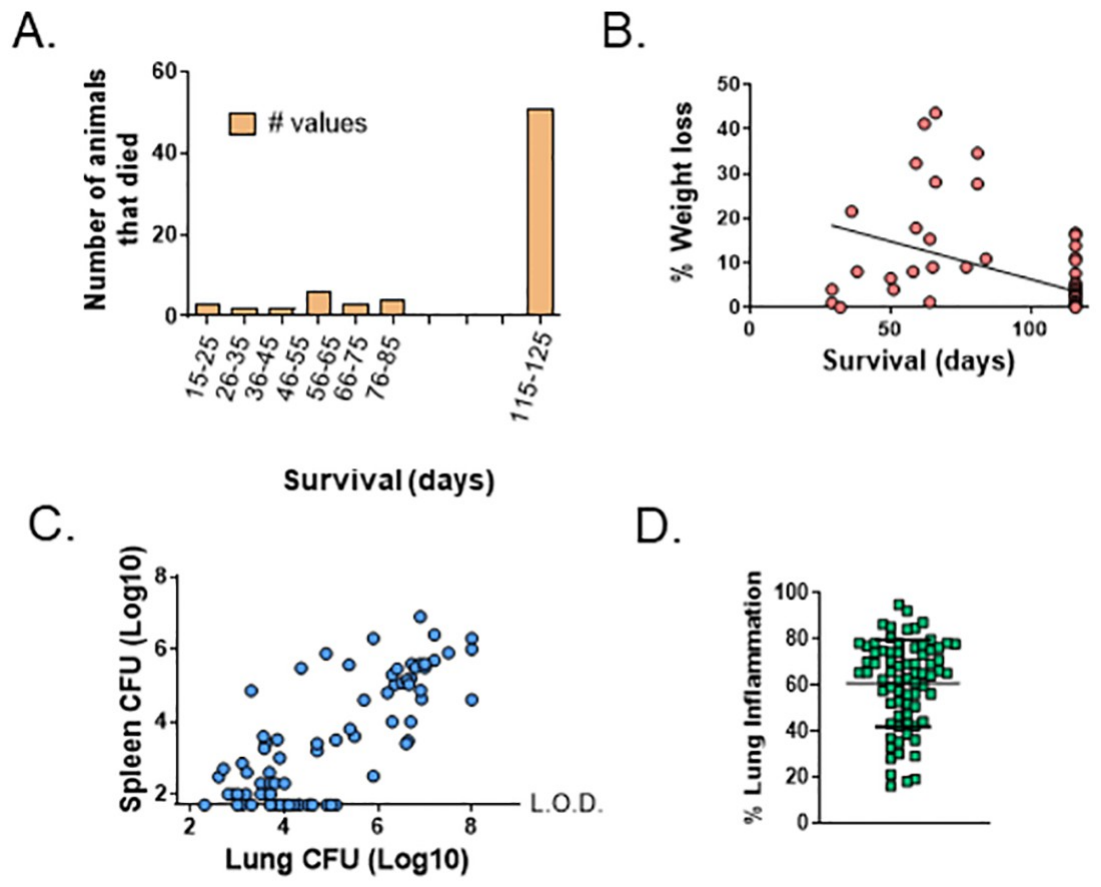
Heterogenous outcomes following BCG-vaccination/*M*.*tb*. challenge in DO mice selected for these studies. From a larger study of ~ 1000 DO mice, animals were ranked based on lung and spleen CFU and survival; for the present analyses, animals with the highest and lowest CFU burdens, as well as those and that succumbed to infection before 14 weeks early, were chosen. Outcomes are illustrated for the 107 female DO mice included here that were vaccinated with 10^5^ CFU of BCG, challenged aerogenically with *M*.*tb*. 8 weeks after vaccination, and monitored for survival through 14 weeks. Animal weights were collected weekly throughout the experiment. Lungs and spleens were processed and plated to enumerate *M*.*tb*. CFU from animals that survived through 14 weeks or that were humanely euthanized prior to 14 weeks due to early morbidity. A lobe was taken from each mouse lung at necropsy and fixed in formalin. Tissues were sectioned, slides were prepared, and slides were H&E stained for histopathological analyses. The proportion of lung tissue with disease involvement was quantitated by densitometry. A) Number of animals that succumbed to infection or were euthanized within each time period. B) Weight loss of each animal as determined by the final body weight/peak body weight. C) Spleen and lung *M*.*tb*. burdens for each animal. D) Proportion (%) lung inflammation with mean and standard deviation across the DO population.

Success with the initial screen using C57 mice justified proceeding to validation of transcriptional correlates candidates in DO mice. DO mice are genetically unique individuals, and therefore we tracked all samples and data individually, with each mouse serving as its own control. Because of the limitations of small blood volumes available and constraints on repeated samplings of individual animals to maintain mouse health, we chose two time points for transcriptional analyses. We obtained blood via the tail vein for the purposes of transcriptional analyses 2 weeks before and 2 weeks after intradermal vaccination with 10^5^ BCG Pasteur, a time point based on the transcription patterns observed in C57s. BCG-vaccinated mice were aerogenically challenged with *M*.*tb*. 8 weeks after vaccination and were followed for survival through 14 weeks after vaccination [28]. The DO mice used for transcriptional analyses are a subset of animals that were a part of a larger study designed to map murine genes associated with infection outcomes (Kurtz et al [unpublished]). A total of 107 female DO mice representing high and low CFU and survival outcomes after challenge were chosen for analyses (see Materials and methods). The majority of mice survived through 14 weeks after infection, with a small proportion of animals succumbing early before seven weeks (6%) or between 7 weeks and 14 weeks (20%; Fig 6A), consistent with previous observations [28]. Mice were weighed throughout the course of the infection, and weight loss in this group of mice significantly correlated with survival (p < 0.001, Fig 6B). We euthanized surviving mice that remained at 14 weeks and analyzed lung and spleen *M*.*tb*. burdens. *M*.*tb*. organ burdens were also obtained from eight moribund mice that required humane euthanasia before 14 weeks. As previously observed [28], a wide range of amounts of *M*.*tb*. were found in lungs and spleens of individual mice; in some animals, *M*.*tb*. burdens in both organs were low, while some mice exhibited high *M*.*tb*. burdens in lungs and spleens, and some mice had disparate outcomes between organs (Fig 6C). Across the population, lung and spleen CFU were significantly correlated with each other, but outliers existed, with little or no relationship between lung and spleen *M*.*tb*. burdens (Pearson correlation = 0.7884, *p* <0.0001). We collected a lobe of each lung at necropsy for histological analyses. H&E-stained slides from the lung sections were scanned, and densitometry analyses were performed to quantitate the proportion of lung with disease involvement/inflammation. Just as there was heterogeneity in the quantity of *M*.*tb*. present in the lungs, the proportion of inflamed lung tissue was also heterogeneous (Fig 6D).

**Fig 7:**
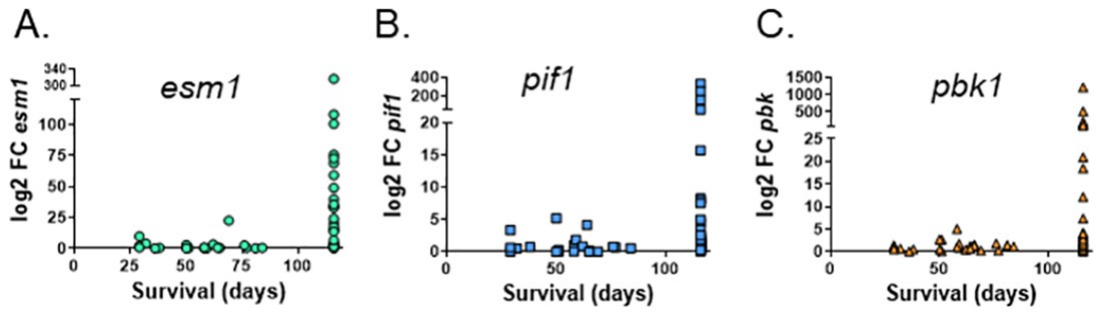
Expression of a subset of correlates genes increases after vaccination in outbred mice. Blood was collected by the tail vein 2 weeks before and 2 weeks after vaccination for each of the selected 107 DO mice (Fig 6) and was processed for RNA isolation. A subset of 11 genes were chosen from the C57 correlate gene panel for analyses in the DO blood RNA (S1 Table). Gene expression was determined by qRT-PCR using gene-specific TaqMan primers/probes, and each gene was normalized to a housekeeping control gene. Gene expression was determined in each pre- and post- vaccination sample, and the log2 change in the fold expression between post/pre vaccination was calculated. Data from the genes A) *esm1*, B) *pif1*, and C) *pbk1* are presented, where each dot represents the value from an individual mouse, linked to the survival of that mouse on the X-axis.

These data were then incorporated into downstream analyses to assess the ability of these gene classifiers to predict outcomes after BCG vaccination/*M*.*tb*. challenge in 107 individual DO mice. We isolated RNA from the whole blood samples obtained before and after vaccination and performed qRT-PCR to measure the transcription of a panel of genes detected as potential immune correlates in C57 mice (Table 2). Because small volumes of blood available from individual DO mice limited RNA analyses, we focused on the 11 strongest candidates of the 18 genes initially detected (Table 1). The log_2_ fold change between pre- and post-vaccination samples was calculated for each gene, and statistical analyses were performed. The differential expression of *esm1*, *stil*, and *pbk* between pre- and post- vaccination samples was readily observed, especially the change in expression between animals that survived through the end of the experiment at 14 weeks and those that succumbed to infection early (Fig 7). Six genes were significantly upregulated between pre- and post- vaccination blood samples as demonstrated by statistical comparisons in Table 2. The relationship between the differential expression of each gene and disease outcomes at 14 weeks after BCG-vaccination/*M*.*tb*. challenge was analyzed. No level of individual gene expression was significantly correlated with the degree of lung inflammation. Differential expression of *stil* or *pbk* was modestly correlated with lung CFU but was not significant. However, *stil* differential expression was significantly correlated with spleen *M*.*tb*. burden (Table 2).

**Table 2:** Relative gene expression of a subset of single-gene classifiers correlates with outcomes after BCG-vaccination/*M*.*tb*. challenge of DO mice.

**A.**	**Differential POST-PRE expression**									
	**All mice**									
	**POST-PRE expression**		**Survival**		**Lung CFU**		**Spleen CFU**		**Lung Inflamm.**	
**Gene**	**Diff.**	**p-val.**	**Diff.**	**p-val.**	**Corr.**	**p-val.**	**Corr.**	**p-val.**	**Corr.**	**p-val.**
***esm1***	0.578	0.235	***2*.*266***	***0*.*004***	-0.117	0.368	-0.089	0.489	-0.07	0.628
***cxcl9***	***1*.*362***	***0*.*018***	1.565	0.182	-0.167	0.201	-0.180	0.163	-0.054	0.709
***gbp2***	***0*.*895***	***1*.*19E-06***	0.347	0.129	0.102	0.346	-0.036	0.741	0.122	0.299
***cxcl10***	-0.516	0.147	-0.906	0.635	0.131	0.446	0.186	0.278	0.021	0.906
***gzmk***	***0*.*916***	***4*.*29E-05***	0.493	0.346	-0.070	0.558	-0.098	0.406	0.040	0.757
***ifng***	***1*.*076***	***5*.*09E-05***	1.003	0.147	0.117	0.295	0.094	0.400	-0.087	0.476
***irx3***	0.423	0.404	0.049	0.878	-0.127	0.406	0.068	0.654	-0.077	0.658
***notch3***	***1*.*430***	***0*.*0003***	-0.125	0.937	0.013	0.920	-0.037	0.780	-0.019	0.896
***pbk***	0.257	0.975	-0.122	0.796	0.215	0.051	0.116	0.292	0.134	0.264
***pif1***	***-0*.*774***	***0*.*038***	1.083	0.22	-0.010	0.934	0.086	0.505	0.017	0.907
***stil***	0.324	0.303	-0.273	0.457	0.198	0.087	***0*.*268***	***0*.*019***	0.12	0.267
**B**.	**Expression post-vaccination**									
	**All mice**									
	**Lung CFU**		**Spleen CFU**		**Lung Inflamm.**		**Survival**			
**Gene**	**Corr.**	**p-val.**	**Corr.**	**p-val.**	**Corr.**	**p-val.**	**Diff.**		**p-val.**	
***esm1***	0.036	0.7626	-0.013	0.9087	0.159	0.2182	***2*.*125***		***0*.*0044***	
***cxcl9***	-0.031	0.7876	0.001	0.9957	-0.191	0.1271	1.101		0.0912	
***gbp2***	0.161	0.1379	0.147	0.1734	***0*.*234***	***0*.*0453***	-0.198		0.5062	
***cxcl10***	0.118	0.4917	0.034	0.8433	0.187	0.2835	-0.332		0.7452	
***gzmk***	-0.017	0.8824	0.123	0.2800	0.131	0.2939	***1*.*146***		***0*.*0431***	
***ifng***	0.004	0.9676	0.066	0.5574	-0.135	0.2622	0.010		0.8309	
***irx3***	-0.057	0.7095	0.023	0.8775	0.267	0.1254	-0.247		0.9204	
***notch3***	0.138	0.2564	0.065	0.5903	0.129	0.3276	-0.526		0.3120	
***pbk***	***0*.*343***	***0*.*0018***	***0*.*234***	***0*.*0319***	0.214	0.0718	0.200		0.6032	
***pif1***	-0.074	0.5436	-0.091	0.4536	0.050	0.7091	0.942		0.1314	
***stil***	***0*.*252***	***0*.*0234***	***0*.*219***	***0*.*0476***	0.161	0.1811	-0.922		0.1952	

Eleven of the correlate genes defined in C57 mice were amplified using whole blood RNA samples from DO mice collected 2 weeks before and 2 weeks after vaccination. Gene expression data were subjected to statistical analyses to determine the differences in gene expression in blood samples collected after and before vaccination, as well as the differences between survivors and non-survivors. For the former analyses, data were transformed into the differential POST-PRE expression, defined as log_2_ expression after vaccination–log_2_ expression pre-vaccination, where negative values were set to 0. The correlation between gene expression and infection outcomes in DO mice were then calculated. Correlations were determined using either the “Differential POST-PRE expression” or the normalized expression of the gene as determined after vaccination (Expression post-vaccination). A) Differences (Diff.) and Spearman’s rank correlations (Corr.) and *p*-values (p-val.) were determined for the change in gene expression before and after vaccination alone (POST-PRE expression), or between the change in gene expression and survival, lung CFU, spleen CFU, and the percent lung inflammation. B) Differences (Diff.) and Spearman’s rank correlations were also determined for the gene expression levels after vaccination and survival, lung CFU, spleen CFU, and the percent lung inflammation. Differences and correlations where *p* < 0.05 are provided in bold/italics text.

Several mice had high baseline (pre-vaccination) levels of gene expression as well has high levels of post-vaccination gene expression, and as a result the differential gene expression between post- and pre-vaccination levels was low. We therefore examined the ability of gene expression as determined after vaccination alone to predict outcomes after challenge. We observed that *pbk* expression after vaccination significantly correlated with lung and spleen CFU (Table 2). Additionally, *stil* and *gbp2* expression correlated with spleen CFU and lung inflammation respectively. Taken together, the post-vaccination expression levels of several of the single gene classifiers discovered in high-throughput screening in inbred mice were significantly associated with infection outcome metrics in outbred mice.

**Fig 8:**
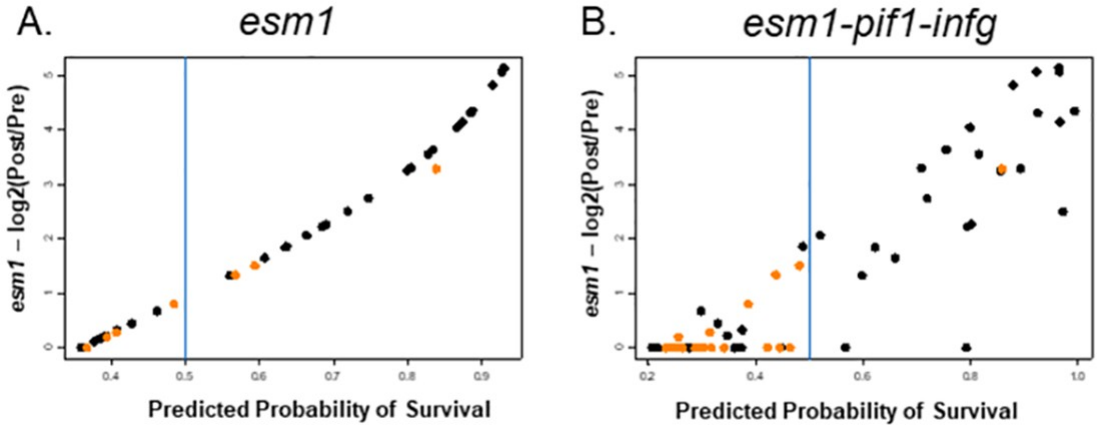
Single and multigene classifiers can predict survival of BCG-vaccinated/*M*.*tb*. challenged DO with high specificity and sensitivity. Modeling analyses were performed with gene expression data from selected DO mice. The predictive probability of survival for individual mice (dots) is presented on the X-axis, with the log_2_ transformed differential expression of the gene *esm1* between post-vaccination and pre-vaccination blood samples (log_2_ POST/PRE) represented on the Y-axis. Orange represents animals that were predicted to be “unprotected” (*i*.*e*., to succumb early). A) The differential gene expression of *esm1* alone predicted survival of the mice with high specificity. B) Combining gene expression data from *esm1*, *pif1*, and *ifng* improved sensitivity and specificity for predicting survival. For A, sensitivity = 0.468, specificity = 0.900 (n = 66 mice). For B, sensitivity = 0.590, specificity = 0.961 (n = 77 mice).

Perhaps the most interesting correlation was between *esm1* and survival. For the purposes of statistical modeling, survival was defined as early morbidity or weight loss greater than 10%, given the relationship between significant weight loss and survival outcomes (Fig 6B). The differential expression of *esm1* alone significantly correlated with survival through 14 weeks and predicted survival of DO mice with high 90% specificity (Table 2, Fig 8A). Measuring *esm1* or gzmk expression directly after vaccination was also highly predictive of survival (Table 2). Combining *esm1* expression with the expression of other genes such as *pif1* or *pbk1* did not significantly enhance the specificity, suggesting these genes may be providing overlapping information in these outbred mice. Sensitivity of predicting death was a modest 46.8%, driven in part by low numbers of animals that succumbed to infection early. Specificity and sensitivity were moderately improved by the addition of other genes, including *ifng* and *pif1*, where 96.2% specificity and 61.5% sensitivity were achieved (Fig 8B). Taken together, therefore, these results demonstrated that transcriptional correlates measured shortly after vaccination can predict long term outcomes after *M*.*tb*. challenge, including survival, in diverse outbred mice.

## Discussion

No tools or immune correlates have been identified in humans to predict efficacy of any vaccines against tuberculosis, even BCG. A variety of approaches, including cellular functional assays of proliferation and cytokine production, flow cytometry-based assays, transcriptomics, and assays of specific antibody binding and functions are under study. Many of these revolve around cytokine production by primed T cells and thus would be applicable weeks or months after vaccination. Here, we focused on finding transcriptional signatures that could be measured quickly after vaccination. We demonstrated that BCG induces informative transcriptional changes in mouse blood and PBL within days after vaccination (Figs 1 and 4, Tables 1 and 2). Unsurprisingly, the transcriptional profile of the initial set of genes screened was variable depending on the sample type and the particular gene, although the expression of most informative genes peaked about 14 days after vaccination (Fig 1B).

RNASeq analyses of transcriptome-wide screening on day 14 provided a set of over 1000 genes that were differentially regulated by BCG vaccination compared to control conditions (Fig 2) and represented a diverse set of biological pathways (Fig 3). The type of genes expressed were consistent with current concepts of protective mechanisms induced by vaccination against TB and supported the relevance of the results. For example, canonical pathway analyses showed differential upregulation of Th1-related signaling, activation, and differentiation (Fig 3A). Memory Th1 CD4 and CD8 T cell responses and related cytokine production, such as IFN-γ, are generally considered to be integral components of protective immune responses to *M*.*tb*. and are stimulated by BCG vaccination [34–36]. The influx of leukocytes and upregulation of genes in cellular development and proliferation pathways are relevant to adaptive immunity, and these responses also align with known responses to BCG vaccination (Fig 3B; [37–39].

To validate initial findings with orthogonal methods, we examined the expression of 94 of the most robust and reproducible gene candidates in a secondary screen. For re-screening experiments, we also added an additional group of animals primed with heat-killed BCG to serve as a comparator; HK-BCG retains some of the stimulatory properties of BCG but does not elicit substantial protective immune responses in mice (Fig 1A; [4]). We focused on identifying genes whose expression patterns followed a protection hierarchy, namely those that were differentially induced by BCG and not HK-BCG when compared to sham-vaccinated animals. qRT-PCR studies, coupled with application of time course and reproducibility criteria, lead to the refinement of a panel of 18 genes with the highest differential upregulation in BCG-vaccinated samples compared to HK-BCG or naïve samples (Table 1). These genes were then subjected to further statistical analyses to determine the ability of each gene to predict the degree of protection afforded by vaccination, *i*.*e*., to serve as a “good classifier,” defined as a classifier with a sensitivity and specificity > 0.7. Of the 18 top candidate genes, when data from whole blood was analyzed, six individually discriminated the BCG-vaccinated group from HK-BCG-treated or naïve groups. Other members of the 18-gene panel could successfully discriminate vaccinate groups when used in concert with other genes from the panel, *i*.*e*., in 2 or 3 gene classifiers (S1 Table). Altogether, each of the 18 genes contributed to at least one good classifier, demonstrating the utility of this working panel of genes for predicting protection.

Our data also demonstrated that gene expression changes were generally most robust early after vaccination at 14 days and waned over time through 56 days; for the four strongest candidates, the expression patterns were similar in blood and PBL (Fig 4). This could reflect the waning strength of stimuli, changes in circulating cell populations, changes in transcriptional responses induced by vaccination, or all combinations. This timing coincides with the expected lifestyle of BCG within the host following vaccination, where BCG initially engages with neutrophils, macrophages, and dendritic cells at the vaccination site [40]. BCG is phagocytosed initially by dendritic cells and can survive within these cells for several weeks, where it triggers DC maturation and migration. BCG is then processed for antigen presentation, which in turn begins the developmental cascade for adaptive immune responses that includes T cell recruitment [40]. The gene expression profiles and biological pathways identified here therefore align with those genes involved with cellular recruitment and cell differentiation during that time frame.

In humans, existing gene signatures are largely used as triage tests, for the purposes of diagnosis or for prediction of progression of disease, including transition from latent to active TB. To date, more than 25 human gene signatures have been proposed for the diagnosis of TB disease or progression of disease in patients [9]. A meta-analysis of these human signatures and found that at least one met the minimum criteria for prediction performance that was set for Target Product Profiles for tuberculosis diagnostic products [9]. In contrast to many studies of transcriptional signatures related to disease status, few studies have reported transcriptional signatures related to vaccination. Here, we focused on the development of a panel of transcriptional biomarkers that can be used to predict tuberculosis vaccine efficacy quickly after BCG vaccination. Of the 18 genes in a vaccination-related panel established using inbred C57 mice, three are found in these various human gene signatures related to disease progression. These include *gbp2*, *cxcl10*, and *gzmk*; one human signature contained all three, a 312-gene signature described by Berry *et al*. [9, 12]. These human signatures were derived using varying methods and study cohorts, and for various purposes. The detection of even this amount of overlap between the genes within the disease-related human signatures and genes from a panel related to vaccination is notable, and this result further supports the relevance of exploring this approach using mice. Moreover, one recent publication demonstrated the utility of developing transcriptional immune correlates across species, in which a human 16-gene signature was tested as a predictive indicator of *M*.*tb*. disease progression in mice and NHPs [11]. This study also uncovered a role for *gbp2* and related family members in resistance to *M*.*tb*. primary infection, further supporting the importance of this factor as a potential predictor of vaccine-induced protection. The signatures defined here, derived from easy to sample whole blood, may represent the potential for establishing easily measurable transcriptional correlates of protection.

The initial screening using C57 mice enabled selection of a time point for blood collection in DO mice shortly after vaccination that would likely reveal robust transcriptional expression. Moreover, screening identified a reasonable number of genes that could be evaluated in a large number of mice. Because we wished to obtain blood samples from the same animal without affecting its health before challenge, we were restricted to obtaining one pre-vaccination bleed and one post-vaccination bleed. Therefore, based on the time course data in C57 mice, we chose 14 days after vaccination as the optimal time point most likely to provide peak gene expression in the blood. We chose to test this correlate panel in ~110 animals to achieve a representative sample size in this heterogeneous population; this size group would be prohibitively large for a primary screen but was feasible for performing targeted validation. We chose 11 of the most robust and reproducible genes from the C57 panel for analyses in the DO mice. Even in the face of heterogenous genetic backgrounds that are quite distinct from the gene pool in C57 mice, the expression of 6/11 genes tested was significantly upregulated after vaccination. In particular, the differential expression of *esm1* alone predicted survival with high specificity (Fig 8A). Also of note, 43 out of 77 mice had log-ratios = 0 (*i*.*e*., very weak *esm1* signals), and among them 19 survived (Fig 8A). Some of these animals may therefore have been mis-classified due to low overall expression signals.

Nonetheless, the 47% sensitivity of *esm1* for predicting early death was moderate. Combining *esm1* expression data with that of other classifiers such as *ifng* and *pif1* improved the sensitivity and specificity for predicting death (Fig 8B), illustrating the potential for multi-gene signatures to achieve useful sensitivity and specificity values. One confounding variable was baseline *esm1* expression before vaccination; animals with high baseline *esm1* expression were less likely to have strong upregulation following vaccination and therefore were classified as likely to die yet survived. This may suggest that *esm1* expression has a biological threshold such that either high baseline expression, or high induction by vaccination, are sufficient to result in levels of *esm1* that predict protection. Indeed, expression of *esm1* measured directly after vaccination alone was significantly correlated with survival, supporting this idea. Also of note, two animals classified as survivors succumbed to early *M*.*tb*. infection. Rare mutations have been observed in DO mice at a higher frequency than in humans [41], and thus it is possible these two mis-classified animals had additional underlying factors involved in their deaths. Of two mis-classified animals, one had moderate levels of *M*.*tb*. in lungs (6.3 log_10_ CFU) and spleens (4.0 log_10_ CFU) at necropsy, levels often observed in surviving mice. *M*.*tb*. organ burdens were unavailable for the other animal.

*Esm1* therefore emerged as a novel and leading candidate as a predictive correlate. *Esm1*, or endocan, is an endothelial cell marker that is strongly upregulated by inflammatory cytokines such as TNF-α. *Esm1* regulates cell migration and inflammation, important in the early development of protective cellular responses [42]. Previously, investigators studied *esm1* in the context of using the gene as a diagnostic tool for tuberculous pleural effusion, but to our knowledge no other reports suggest a direct link between the functions of *esm1* and immune responses to *M*.*tb*. [43]. This is an obvious area for future study, as are other unexpected and unique candidates. For example, *Irx3* (Iroquois homeobox 3) was also highly differentially expressed by day 14 (Fig 4). *Irx3* belongs to a superfamily of transcription factors that can act as either repressors or activators of gene expression ([44, 45] and is commonly linked with embryonic development of neuronal and cardiac tissue. *Irx3* is also associated with malignancies in humans, such as particular subtypes of leukemia, where it may influence T cell maturation checkpoints [46, 47]. While the role that *irx3* plays in immunity to *M*.*tb*. has not been directly studied, it is tempting to speculate that *irx3* may similarly be involved in regulation of T cell differentiation in response to BCG vaccination.

Other candidates identified in this unbiased screen have plausible links with immunity to tuberculosis. These include *Cxcl9* and *cxcl10*, which both produce ligands that bind to the common receptor CXCR3; receptor binding in turn stimulates cell migration, including migration of Th1 T cells. Following *M*.*tb*. aerosol infection of Rhesus macaques, CXCR3^+^CD4^+^ cells in lungs were inversely correlated with *M*.*tb*. burden [48], suggesting the importance of these cells in appropriate homing to respond to infection. Granzyme K (*gzmk*) belongs to a family of granule-secreted serine proteases produced by T cells and natural killer (NK) cells. GzmK has a variety of roles in host-pathogen responses that include functioning as a pro-apoptotic protein and promoting endothelial cell activation and pro-inflammatory immune responses [49]. *Gzmk* expression was upregulated in lungs following *M*.*tb*. infection in mice [50]. In humans, changes in *gzmk* expression were observed in comparisons of whole blood transcriptional signatures between patients with active versus latent TB or in PBMCs from healthy household contacts and LTBI or TB patients [51, 52]. While other related granzymes *gzmA* and *gzmB* have been studied for their contributions to anti-mycobacterial immunity [53, 54], the role of *gzmK* in the host response to *M*.*tb*. has not yet been directly tested. Although the main goal of this study was to assess the potential for developing a transcriptional signature that predicts vaccine-induced protection, future studies will also seek to understand mechanistic roles that in turn may suggest the most robust candidates to retain in signature panels. Future studies will also aim to assess the applicability of our signature, derived from studies using live BCG, to other vaccine platforms, such as subunit or viral-vectored vaccines. As yet, it is unclear whether common or even universal signatures of protection may be uncovered, or whether any given vaccine platform will utilize different pathways to protection and therefore would elicit unique gene signatures.

Taken together, these studies yielded a panel of genes whose expression in blood soon after primary vaccination can serve as transcriptional immune correlates for vaccine-induced protection against *M*.*tb*. This approach has the appeal of logistical and technical simplicity. Moreover, the panel developed in inbred mice, and tested in outbred mice, is also rooted in the biology of protection. The encouraging results support advancing studies to evaluate the predictive capacity of these working gene signatures in NHP and people.

## Supporting information

S1 Table All single and multigene classifiers included in modeling analyses to predict survival of BCG-vaccinated/*M*.*tb*. challenged DO with high specificity and sensitivity. Modeling analyses were performed with gene expression data from selected DO mice. Each tab includes the day the sample was collected, tissue of origin, accuracy, sensitivity, and specificity for each classifier, and the single or multi-gene components of that classifier. Data are provided for day 14, day 28, day 56, and day 56-restimulated (Day 56RE). A subset of the data are provided in a separate tab which describes all classifiers with a sensitivity and specificity > 0.7.
(XLSX)
